# Absenteeism amongst health workers – developing a typology to support empiric work in low-income countries and characterizing reported associations

**DOI:** 10.1186/1478-4491-11-34

**Published:** 2013-07-17

**Authors:** Alice Belita, Patrick Mbindyo, Mike English

**Affiliations:** 1 Kenya Medical Research Institute -Wellcome Trust Research Programme, Kenyatta National Hospital, Hospital Road, Nairobi, 00100, Kenya; 2 Nuffield Department of Medicine and Department of Paediatrics, University of Oxford, Old Road Campus, Oxford, OX3 7BN, UK

**Keywords:** Absenteeism, Workforce, Sickleave

## Abstract

The contribution of inadequate health worker numbers and emigration have been highlighted in the international literature, but relatively little attention has been paid to absenteeism as a factor that undermines health-care delivery in low income countries. We therefore aimed to review the literature on absenteeism from a health system manager’s perspective to inform needed work on this topic. Specifically, we aimed to develop a typology of definitions that might be useful to classify different forms of absenteeism and identify factors associated with absenteeism. Sixty-nine studies were reviewed, only four were from sub-Saharan Africa where the human resources for health crisis is most acute. Forms of absenteeism studied and methods used vary widely. No previous attempt to develop an overarching approach to classifying forms of absenteeism was identified. A typology based on key characteristics is proposed to fill this gap and considers absenteeism as defined by two key attributes, whether it is: planned/unplanned, and voluntary/involuntary. Factors reported to influence rates of absenteeism may be broadly classified into three thematic categories: workplace and content, personal and organizational and cultural factors. The literature presents an inconsistent picture of the effects of specific factors within these themes perhaps related to true contextual differences or inconsistent definitions of absenteeism.

## Background

There is a human resources for health crisis in sub-Saharan Africa [1-4]. The health worker density in most sub-Saharan countries is well below the WHO recommended minimum of 2.5 health workers per 1000 population [1,5] while the burden of disease is high [1]. One consequence of low health worker density is relatively poor health outcomes of the population [6,7]. Health worker availability is undermined by the hiring freezes present in some countries in sub-Saharan Africa [8-10], and ‘push’ and ‘pull’ factors promoting emigration from low-income countries (LICs) to high-income countries (HICs) [11,12]. Similar factors also create an uneven domestic distribution of health workers, which especially affects rural areas [13] and may promote moonlighting and absenteeism, among other problems [7,14,15]. However, relatively little attention appears to have been paid to absenteeism as a cause of poor access to health-care services in low-income countries.

We therefore aimed to review the literature on absenteeism to inform potential work on this topic in a low-income setting. Specifically, we aimed to develop a typology of absenteeism definitions and identify factors reported to influence absenteeism among health workers.

## Methods

Although our primary interest was absenteeism in low income countries an initial screen of the literature revealed very few studies specific to these settings. We therefore included in our search work from all settings. Potential articles for inclusion published in English between January 1982 and May 2012 were identified through searches of: PubMed, Web of Science, CINHAL and The Cochrane Library. The search terms used were ‘absenteeism’ and ‘health workers’; their respective MeSH terms were also incorporated in the search. No active search of the grey literature was undertaken after initial searches yielded a substantial number of peer-reviewed articles. Additional articles were however sought by screening the reference lists of identified studies and reviews.

For the purposes of this review, we took a health system manager’s perspective. Thus we did not specifically seek to explore deeper psychological or social aspects of absenteeism. Nor did we aim to examine it from an economic perspective. The framework or ‘lens’ though which we explored the literature aimed to uncover how absenteeism has been defined, usually for the purposes of quantification at some level of the health system e.g. within a facility, a region or a profession. Additionally, we sought to elucidate characteristics of the workforce likely to be familiar to managers that have been associated with absenteeism. We acknowledge that such characterization may not explain why health workers are absent. Alternative theoretical frameworks might be needed for such explorations. However, we reasoned that providing both a typology of absenteeism and a description of characteristics associated with it would be a good starting point for developing better informed quantitative and qualitative work in this area in the future.

Eligible studies and reviews were those that reported data on definitions and determinants of, or associations with, absenteeism among health workers. Studies of multiple cadres of hospital staff (e.g. administrative, cleaning staff) were included if they also included doctors and nurses. Article selection was performed by one reviewer who screened through the titles and abstracts and identified and selected studies based on the pre-defined study eligibility criteria. Where there was uncertainty over inclusion of a study, a second investigator was consulted and a consensus decision reached. Key aspects of all included studies, such as population studied, methods, etc., were documented in a standard format to facilitate later summary and synthesis (see Additional file 1).

After abstracting data on each manuscript we assigned the definition(s) of absenteeism employed by authors into initial groups based on similarities allowing, where necessary, reported definition(s) to be assigned to more than one group. In an iterative process thematic groupings that would capture all definitions were developed with the aim of providing a relatively parsimonious classification system and one that captured notions of expected or approved absence on the part of an organization and individual volition. In a similar way, factors reported to be associated with absenteeism were first identified within individual studies, then grouped and then assigned to thematic categories.

## Results

**Figure 1:**
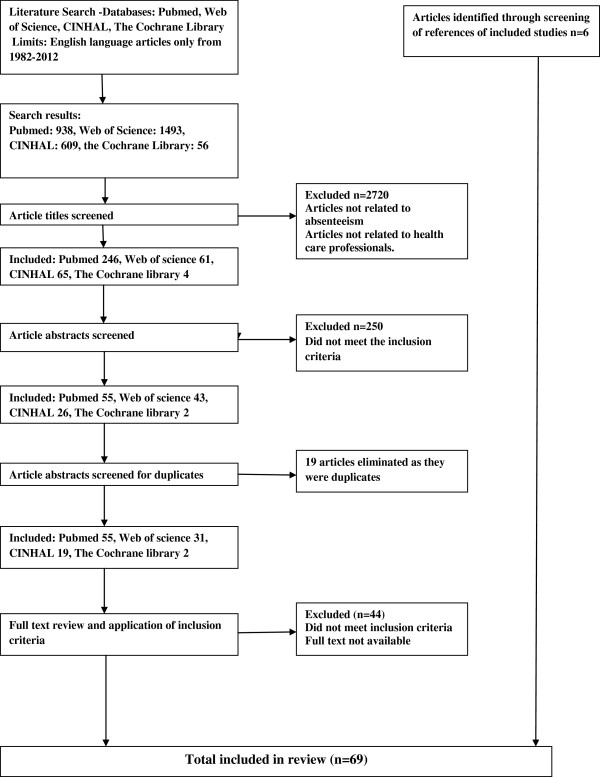
Flowchart of study identification and inclusion.

A total of 3096 studies were identified in initial searches of all the databases. After reading through the titles and abstracts, 107 studies were further screened based on the full text with 63 studies retained for inclusion in the review. An additional six studies were identified through screening of the references of included studies. This process is summarized in Figure 1.

Included studies focused on nurses (n = 36), all hospital workers including administrative and cleaning staff (n = 25), physicians (n = 1), radiographers (n = 1), dentists (n = 1) and combined nurses and physicians (n = 5). The predominant study designs were surveys based on either retrospective or prospective analysis of absenteeism data obtained from administrative records. Other study designs used were retrospective and prospective cohort studies or studies based on interviews, focus group discussions, case control analysis or literature reviews. The majority of studies available were from HICs. Of the seven studies from low- and middle-income countries (LMICs) relevant to this review, four were conducted in Africa.

### Definitions of absenteeism

Definitions of absenteeism were reported in most of the articles reviewed. Some definitions were narrow and characterized absence by specific health conditions, such as knee and back pain [16-18], or stress [19], etc. Other definitions were broad and studied absence in general, typically including absence due to illness and other causes [20-22]. Although actual definitions varied, a typological framework could be established using the following classifications of absence: planned or unplanned, voluntary or involuntary as illustrated in Table 1. Typically such classifications are, however, based on health worker reports of the reason for absence, the veracity of which is often not determined [23].

**Table 1:** A typological framework for defining absenteeism with examples of the different forms of absenteeism

	**Planned**	**Unplanned**
**Voluntary**	Statutory absence (annual/ vacation, study, maternity, off-duty leave), training, workshops, conferences [22,24-26]	Sickness absence to attend to personal matters as they arise but reported as minor illness, often short-term self-certified [26,35]
		Failing to report to work and not giving a valid and acceptable reason for one’s absence (e.g. moonlighting) [26,35]
**Involuntary**	Long-term sickness e.g. ≥90 days in Scandinavian countries [27]	Transport problem, taking care of a sick child/relative, personal injury, sickness that is medicallycertified [52]
	Absence caused by social obligation rather than for personal interest/benefit e.g. attendance at pre-specified event such as a political or community meeting. In such cases, a different allowable reason for absence may be provided to the employer in order to get time off [35]	

The typology outlined draws on ideas presented across the reviewed literature. Beil-Hildebrand [23] established that nurse absence can be divided into planned and unplanned absence. Planned absence occurs when both the employee and employer are aware that the employee will not be coming to work and hence are able to plan in light of that awareness. Different examples of planned absence that fit under this definition have been studied [22,24-27]. Unplanned absence, on the other hand, occurs when an employee does not go to work and the employer actually expects him/her to be at work [23]. Sickness absence is the most common form of such absence studied (for examples see [28] and [16,29-32]).

Perhaps more subjectively, absence may occur for reasons officially allowed or not allowed as per the organization’s policies. Sanctioned absence might entail different forms of allowed time off such as annual, maternity [26], or sick leave, workshop or seminar attendance [22], or approved compassionate leave to take care of sick relatives [33]. Unsanctioned absence might involve health workers not coming to work and either not seeking or not obtaining official approval for such absence. In their study to establish the effects of organizational changes in health facilities in Costa Rica, Garcia-Prado and Chawla [34] examined absence of this type that was either unjustifiable or unexplained. Gaudine and Gregory [35] in their study used questionnaires and asked nurses to report how manydays they were away due to sickness, stress or to attend to family and personal matters. They regarded non-sickness absence as forms of ‘unsanctioned absence’ [35]. However, further subclassification of absence into sanctioned or unsanctioned forms may not always be clear and at present this categorization is not formally included in the proposed typology.

Absenteeism can also be classified as involuntary absence occurring for reasons beyond the employees’ control and voluntary absence occurring when the employee makes the decision not to go to work [36]. It has been acknowledged that differentiating between voluntary and involuntary absence can also be difficult. However, assessment of frequency and duration of absences/sickdays has been used to distinguish between the two. High frequency of absence has consistently been regarded as a reliable measure of voluntary absence [36]. It has further been suggested that unplanned absence is often short-term and sometimes voluntary [23]. Borda and Norman [37] in their study of nurses in Malta considered one-day or two-day absence frequency as a measure of ‘avoidable’ absence and related to job satisfaction, which was of concern in their study. Voluntary absence can also be planned as in the case of maternity [26] and annual [24] leaves.

It is clear that absence, even with availability of attendance records, is not always easily defined or measured. In LICs, attendance records may not be easily available [34] and may lack the required data or detail. Use of self-reported absence is often therefore demanded [38] but may be subject to bias. Determining the actual nature of the absence is thus challenging. For these reasons, if self-reported absence is used as an approach, it may be useful to explore real examples of absence and classify these posthoc. The typology presented could then be used to help classify forms of absence and their relative importance.

### What influences absenteeism? A system perspective

Different factors were reported to influence health workers’ absence. Broadly, these factors fitted into three thematic categories; workplace/content, personal and organizational/cultural factors. They are discussed in detail below.

### Workplace factors

#### Employment sector

Absenteeism has been reported to be common in the public sector in high- and low-resource settings. Garcia-Prado and Chawla [34] in an evaluation of reforms in the Costa Rica health sector noted that absenteeism is rampant in public organizations, although it is often not addressed in policy frameworks in the health systems of LICs [21,34]. Garcia-Prado and Chawla attribute absenteeism in public institutions to the fact that employees get their salary irrespective of performance [34]. This phenomenon has also been observed in HICs as reported by Johnson et al. [39] in the UK and other developed nations [40].

#### Size of an organization

The size of an organization has been said to influence absenteeism [41]. The argument being that big organizations have less group cohesiveness, greater bureaucracy and that individual efforts go unnoticed [34]. Garcia-Prado and Chawla’s work in Costa Rica [34] evaluating the impact of changes in reimbursement methods and organizational reforms on absenteeism found that absenteeism generally increased, and more so in big hospitals than small hospitals. In Kenya, Muthama et al. [42] reported that health workers in district and subdistrict hospitals were more absent than those in health centres and dispensaries.

#### Facility location

The location of the health facility, i.e. whether rural or urban and also in relation to where the health workers live has been reported to influence the absence rate of the health workers. Muthama et al. [42] hypothesized that absenteeism would be greater in rural areas linked to irregular transport and health workers needing to travel long distances to access banks and other facilities, factors associated with absenteeism in Nigeria [38]. However, they found that facilities in urban areas had higher absenteeism [42]. In Bangladesh and Kenya, health workers that lived in the same town or village as the health centre they worked in were less absent compared to those that lived away from their place of work [21,42].

### Work content factors

#### Workload

Heavy workload has been identified as a reason for both short- and long-term sickness absence. This may encompass means to escape this heavy workload or to recover from illnesses caused by managing the heavy workload [43-46]. As with many factors influencing absenteeism, effects are not always consistent. Kivimaki et al. [44] in their study on physicians’ sickness absence in Finland found that feeling overloaded increased the risk of short spells of absence in male physicians and the risk of short and long spells of absence among head nurses and ward sisters. Feeling overloaded, however, did not affect sickness absences among female physicians [44]. In Canada, a study among physicians on the impact of heavy workload on their attitudes and outcomes found that absenteeism increased with increase in workload [19]. Similar trends were observed in studies among nurses and health-care workers [47-49]. Using the RAFAELA™ (a human resources management system) patient classification system Rauhala and colleagues [50] found that individuals exceeding the optimum workload by 15% or more had increased risk of sickness absence. This association was the same for both short-term (self-certified) and long-term (physician-certified) sickness absence [50].

#### Working conditions

Effects of working conditions can be related to structural or organizational aspects of work and social aspects. In the United States of America, Trinkoff [51] found that nurses in jobs where they worked with their head or arms in awkward postures were significantly more likely to be absent than those without such demands. In Sweden, Josephson et al. [43] found that nurses dissatisfied with the quality of care provided to patients had higher probability of being on long-term sickness absence. Interference with safety requirements was reported to expose Costa Rican health workers to work-injury absence [52]. Work schedules and terms of contract have been identified as factors that could influence a health worker’s presence or absence from work. Ritchie et al. [53] established that part-time staff in the UK had lower rates of absence than full-time staff among most occupational groups, similar to findings from Canada [54-56].

The social context of work is also important. Kivimaki and colleagues [57] found that Finnish health workers that had experienced social exclusion, also seen as a form of bullying, had a high rate of absence. Similarly, bullying and violence from colleagues, patients and visitors was reported to increase absenteeism among nurses in Sweden [43], the USA [58], the Philippines [30], Canada [59], Turkey [60] and radiographers in Hong Kong [61]. Support from colleagues and supervisors may also be influential. In Finland, doctors with poor teamwork were reported to have long sickness absence spells [44], while nurses in primary care were more often absent than those working in teams [62]. The relationship between absenteeism and job satisfaction is inconsistent. Albion [63] reported that high levels of workplace distress among health professionals in Australia were associated with greater duration of absence. Job satisfaction influenced absenteeism in some studies [64,65] while in others there was no apparent relationship [66,67].

#### Organizational changes

Changes in organization of health facilities may increase absenteeism depending on how the health workers perceive them [40,43]. In Costa Rica, a study was done to assess the effect of hospital management reforms on absenteeism in 29 public hospitals. It was found that introduction of the reforms, which among many other objectives aimed to reduce absenteeism among health workers, actually resulted in an increase in absenteeism [34]. This was attributed to union resistance to the reforms [34].

### Individual level factors

Individuals’ personal characteristics influence the absence rate, duration, and reason for absence [56,68].

#### Marital status

Marital status has been reported to influence absence from work in different ways in different settings. In Finland, Kivimaki et al. [44] found that male physicians, head nurses and ward sisters who were married were absent less. Absence of female physicians was, however, not influenced by their marital status. In contrast, married Nigerian health workers were reported to be more absent and, of the reasons given, dealing with family problems was common [38]. Borda and Norman [37] noted that family responsibilities increased the probability of female nurses being absent while work-family conflict among Swedish nurses increased the odds of one resigning or being on long-term sickness absence [43].

#### Age

In their study in India, Tripathi et al. [26] found that unplanned sickness leave rates were highest among older ward nurses while the highest planned sickness leave rates were among younger operating theatre nurses, with absence mainly attributable to childbirth. In Sweden, nurses older than 50 years had a higher rate of long-term sickness absence [43] with similar findings in Nigeria [38], Canada [56], and Finland [44]. However, increase in absenteeism with increase in age [68] is not always found [55]. Trinkoff et al. [51], while studying effects of physical demands among registered nurses in the USA, established that younger nurses took more sickdays. Such findings are also reported from Nigeria where retrospective analysis of sickness absence records of all hospital employees also found that younger employees and those employed for shorter periods of time had a higher rate and duration of absence [69].

#### Gender

Most studies report that women are more often absent than men [68,70-72]. A study among all employees of four National Health Service trusts in Scotland reported that women were more likely to have been absent and had higher absenteeism rates across all professions [53]. In Nigeria, a study using self-reported absence and incorporating all hospital staff also established that women were more absent than men [38]. Similar findings have been seen in British Columbia [56], Finland [44], and Sweden [43]. However, Bamgboye and Adeleye [69] in a different study in Nigeria among all hospital employees and Chaudhury and Hammer [21] in their study in Bangladesh among health workers did not find any significant difference in absence rates between men and women.

#### Level in the hierarchy

Among health workers, cadres that are higher up in the hierarchy, for example doctors, are said to be less absent compared to other cadres. Kivimaki et al. [44] found that physicians had lower rates of short- and long-term sickness absence compared to the nurses in Finland. In the UK, Ritchie et al. [53] report that auxiliary staff had the highest rates and duration of absence while medical and dental staff had the lowest. Such findings are supported by reports from Nigeria [38,69], Saudi Arabia [73], Thailand [74], Denmark [71] and Switzerland [18].

#### Individual health status

The health of an individual is bound to affect how often and how long they are away from their place of work [16,44,46,56,59,69]. In Sweden and Norway, health workers with self-reported health complaints had increased risk for sickness absence [27,75]. In high-income settings, studies have often focused on musculoskeletal and psychological (stress or burnout) disorders [16,27,43,76]. However, HIV and AIDS infection as a human resources for health problem is mostly unique to Africa [3,12]. HIV and AIDS infection has increased the workload for health workers [77] and led directly to a reduction in numbers and increased sickness absence [78]. Indeed, people that are HIV positive have been reported to be absent from work for up to 50% of the time in their last year of life [78], added to which is the issue of caring for affected relatives.

### Context- or culture-specific reasons

Although there were not many studies on context-specific reasons for absenteeism among health workers, a number of studies implied contextual reasons as associated with absence.

#### Management

The leadership style used in health facilities can influence the absence trends of health workers. In the Netherlands, managers’ leadership effectiveness was inversely related to the number of absencedays and short-term absence of nurses although there was no relationship to long-term absence [79]. However, in work also undertaken in the Netherlands investigating the effects of two leadership styles neither had an effect on absence frequency, although it was proposed that a combination of different styles could lower absence frequency [80].

#### Cultural expectations

The expectations of peers and society at large might influence absence patterns among health workers. Although this was not explicitly stated, it is implied that absence ‘culture’ can influence the absence patterns of health workers [45]. Cultural differences in absenteeism can further be noticed in that some reasons for health worker absenteeism were only found in studies done in LICs. Isah et al. [38] in their study among the entire staff of a hospital in Nigeria identified common causes of absence including: attendance at examinations, social events like marriage and burial, adverse weather conditions, and travel and transportation problems. These are reasons that were not generally reported in studies done in HICs.

#### Policies

In Costa Rica, reforms put in place with the objective to reduce absenteeism actually resulted in an increase in absence [34]. The reforms included management contracts with a sick leave policy of not substituting absentee workers intended to reduce overtime costs and activate a peer pressure mechanism to prevent absence. However, this was hard to implement due to union resistance [34]. In Ethiopia, problems arose after legalization of private health care. Here Lindelow and Serneels [7] in a qualitative study reported that health workers were absent from the public sector while attending to patients in the private sector. There were even cases where health workers referred patients from the public sector to their private practice.

## Discussion

This study confirms that there are varied definitions of absenteeism that depend on the context in which the study is conducted, the availability of informative routine data and the factors investigators consider to be most problematic as causes. Even in the same contexts, varying definitions and measures have been used [27,43]. The variability of definitions often proves a challenge when comparing or contrasting research findings, particularly perhaps when trying to understand the apparently inconsistent findings for factors associated with high rates of absenteeism. Drawing generalizable lessons from the body of literature reviewed was also made more difficult by the need to combine literature from countries at all stages of development. However, although our interest was driven by the desire to understand absenteeism in LICs the paucity of research from these settings precluded this.

Our review suggests that while investigators may study varied and multiple forms of absenteeism they might consider how these could additionally be characterized based on two key characteristics (Table 1) as: planned/unplanned and voluntary/involuntary. Using such a classification system may promote the generalizability of research findings and promote exploration of different patterns of absenteeism across different contexts. Classification systems should ideally be easy to use. Further work in this area may justify the inclusion of a further approach to classification to encompass whether the absenteeism episode was officially sanctioned or not (unsanctioned). For example it is possible that some forms of absence might be unplanned, involuntary and unsanctioned. Such a form of absence might result if health workers absent themselves from their expected station to cover for a colleague who is absent. The primary absence thus results in an indirect secondary absence. It seems intuitive that these forms of absence are possible but may be hard to confirm using the methods that predominate in this field to date that rely on self-reported absence or analysis of administrative records.

Despite difficulties with definitions and very limited literature, there are some forms of absenteeism that appear to be of greater concern in LICs, our particular interest. One form of planned absence is attendance at workshops that takes health workers away from their duty stations [22], another clearly the direct impact of health workers’ ill-health in Africa resulting from the HIV pandemic. A related problem that may be more prevalent in LICs and that our search may not have captured is presenteeism, the notion of being formally present at work but preoccupied with non-work-related activities [44].

However, it also needs to be noted that health worker absenteeism has often been difficult to measure in both high- and low-resource settings [68,69,81]. Absence records in some form in HICs are often routinely available [62,79,82]. Some LICs report having sickness absence records but these often seem to be incomplete or inaccurate [45,69]. Even with availability of records, it remains challenging to verify reasons for absence, especially when reasons are self-certified or self-reported, as false reporting and recall bias may limit meaningful interpretation. Interestingly, in one study of nurses in Canada [35], a strong positive correlation and a strong intraclass correlation were observed for self-reported and administratively recorded absence, although a majority of nurses underestimated their absence [35].

The bulk of studies on absenteeism have been in HICs and mostly among nurses [40,79,83,84]. The reason that investigators tend to focus on nurses is not clear. However, we might speculate that this recognizes that numerically nurses are typically the largest single health worker group, that records on nurse absence may be better kept or that investigators feel less comfortable investigating the historically more powerful group of physicians. Amongst nurses, the work tends to coalesce around sickness absence probably linked to the high cost of such absence to patients, governments, taxpayers and even insurance companies [82,85,86]. Indeed the design of sickness insurance systems and other forms of social insurance may have a major impact on sickness absence [39]. However, sickness absence may in some cases be an expeditious way of avoiding negative work environments or experiences. It may also be the only way to get time off work to attend to other personal matters [26] (in some settings referred to as ‘taking a sickie’ [87]). Since it is sometimes difficult to distinguish between genuine sickness and shirking, frequency of absence has been used as an indicator of voluntary absence [23,36] and is predicted by variables such as job commitment. Long duration of absence on the other hand is often felt to be indicative of poor health and is predicted by variables such as burnout [63] and musculoskeletal injuries.

Cadres higher in the hierarchy have been reported to have lower rates of absence [44,53]. They have also been reported to have higher presenteeism than other cadres [44]. As such studies are few, caution is required when interpreting such findings. It is plausible that professional values that require them to be physically present and a lack of people to cover for them when they are away (as they are fewer in number) [88] might reduce absenteeism. However, it is equally plausible that there is poorer collection of information on absenteeism amongst such cadres [38,69]. Indeed in LICs there is often high demand for the services of the few higher level cadres and reports suggest they may be absent from the public sector while providing services in the private sector, especially where policies are relatively permissive [7,42]. At all levels of the hierarchy absence may be driven by physical job strain and lack of autonomy and satisfaction [26,65].

Probably due to the complexity of health worker absenteeism, studies on interventions to reduce it are few. A Cochrane review on effects of preventive staffsupport only identified one study that aimed to reduce absenteeism [89]. It showed no significant effect of the intervention [20]. Indeed on occasions policies aimed at controlling absence have resulted in higher absence by undermining employee commitment [39] or producing other unintended effects on management or employee behaviour [34].

## Conclusions

Health worker absence in LICs is likely to exacerbate human resources for health inadequacies and undermine demand for, quality of and efficiency of delivery of health services. Despite this it has been infrequently examined by researchers, by far the majority of work having been conducted in HICs. Yet absenteeism may be a barometer for the psychological and physical well-being of health workers and a valuable measure of health systems performance. Further work in LICs might be of more generalizable value if a structured typology of the forms of absenteeism is used. This may help us further understand how different factors related to the work setting, nature of work, individual characteristics, and context influence different forms of absenteeism, thereby providing a rationale for interventions to address it.

## Competing interests

The authors declare that they have no competing interests.

## Authors’ contributions

ME in collaboration with CDC’s Health Systems and Human Resources Team, Division of Global HIV/AIDS, developed the research objective for the review. AB carried out the literature searches, retrieved manuscripts and abstracted key data with the help of PM and ME. AB led the efforts to develop the typology and characterize themes emerging from the literature with the help of PM and ME. AB drafted the initial manuscript with help from ME. All authors read and approved the final manuscript.

## Supplementary Material

Additional file 1 A summary of studies included in the review. Click here for file
