# Supplementary material for: Absenteeism amongst health workers – developing a typology to support empiric work in low-income countries and characterizing reported associations
Source: Hum Resour Health. 2013 Jul 17;11:34. doi: 10.1186/1478-4491-11-34 (PMC3721994; doi:10.1186/1478-4491-11-34)
Supplement: Additional file 1 — A summary of studies included in the review. [file 1478-4491-11-34-S1.pdf]

Summary of studies reviewed

Author, yr, Country	Research Question/ Aim	Respondent Population	Method/ Design	Findings
Andreasson 2001, Sweden	To assess the consequences of work-related skin and airway symptoms among these dentists, in terms of contact with health authorities, sick leave, or changes in professional career.	3082 of 3500 dentists in public and private practice	Questionnaires sent to 3500 dentists.<br>Phone interviews with 19 dentists who reported change of career due to work related skin and airways symptoms.	MSD known to cause sickness absence among dentists.<br>Skin and airways infections did not significantly contribute to sickness absence and change of career.
Albion MJ 2008, Australia	To explore relations among positive and negative psychological states and both absenteeism and turnover intentions.	1097 of 1683 hospital employees were surveyed using the Queensland Public Agency Staff Survey (QPASS)	Survey used to obtain measures of organizational climate, psychological reactions to work, job satisfaction, and self-reported levels of intention to leave.	The hypothesised mediation effect of psychological states on the relationship between organizational climate and absenteeism did not emerge.
Alexopoulos 2011, Greece	To investigate the relationships between physical, psychosocial, and individual characteristics and occurrence, chronicity, care seeking and absenteeism due to musculoskeletal complaints of the lower back and knee.	350 of 448 nursing personnel in six hospitals	A self administered questionnaire was distributed by the researchers between September and December 2007.	Perceived moderate/bad general health and high need for recovery were risk factors for absenteeism due to low back pain.<br>Organizational factors, high job demands and low co workers support were risk factors for absenteeism due to knee pain
Al-Shammari, Bamgboye et al. 1994, Saudi Arabia	To analyse the sickness absence records of medical staff in a university teaching hospital and identify the categories of hospital personnel that are at high risks and causes for sickness absenteeism.	Workers in a hospital (contract and non-contract) including admin and support staff.<br>N=861	The sickness absence records of workers at the King Khalid University Hospital over a period of two years (1990-1991) were analysed to identify the category of workers at high risks.	Prevalence of sickness absence higher among contract workers than non-contract workers.<br>The sex ratio was 1:2 in favour of females among contract employees but 1.2:1 in the male's favour for the Saudis.<br>Females have more spells of sickness than males and the duration of sickness absence is significantly higher in females.<br>No significant occupational effect on the spells and duration of sickness absence.
Anagnostopoulos 2010, Greece	To examine how job burnout was associated with sick-leave	Nurses n=487	Cross-sectional study. Use of questionnaire survey measuring	54% had no sickness absence recorded in the last 12 months, 37.6% had taken

	absence and quality of life		burnout levels and quality of life. These were then matched to absence data from hospital records	up to 10 days, and 8.4% had taken more than 10 days.<br>Significant negative correlations observed between sickness absence duration and mental and physical health<br>Nurses with high burnout levels were considerably more likely to be absent from work for a short period than those with low burnout levels
Bourbonnais 1992, Canada	To test the hypothesis:-That among nurses, the heavier the work load, the more sick leave nurses would take	Nurses from 7 general hospitals in Quebec who had been employed for at least 6 months.	Case-control study<br>Cases (experienced at least one episode of medically certified sick leave for a diagnosis "most likely to be related to work load" between 1.01.1984 and 31.05.1987) n=184, Controls n=1165	Significant associations found between sick leave and nurse patient ratio among head nurses, patient's duration of stay, and one hospital. Sick leave more frequent among full time permanent nurses and among those on night and evening schedules<br>Head nurses more absent than nurses
Bourbonnais 2001, Canada	To determine whether nurses exposed to job strain had a higher incidence of sick leave than nurses not exposed<br><br>To determine if social support at work modifies the association between job strain and the incidence of sick leave among nurses	Nurses n=1891 of 3065 1793 of 1891 consented to examination of absence records	Longitudinal study.<br>Use of questionnaires and examining absence records	Short-term sick leaves associated with job strain and with low social support at work.<br>Certified sick leaves also significantly associated with low social support at work<br>Social support at work does not modify the association between job strain and absence
Bamgboye and Adeleye 1992, Nigeria	To fill the gap in the knowledge of sickness absence records and statistics among Nigerian hospital workers.	Employees in a University Teaching Hospital n=3112.	The sickness absence records of employees in a University Teaching Hospital in Nigeria were examined over a period of three years.	An overall proportion of absentee workers was 15.8% with an average of 3 spells of sickness per year per absentee while the duration of sickness per absentee was 5.6 days per year.<br>The younger employees less than 35 years of age and those with short duration of employment with the hospital have significantly higher spells

				and duration of sickness absence than others.<br>Lower spell of sickness and duration of sickness absence were observed among nurses, senior employees especially doctors had no records of sickness absenteeism in any of the 3 years of study.
Bamgboye, Olubuyide et al. 1993, Saudi Arabia	To determine:<br>(a) The rate of sickness absenteeism among workers of a teaching hospital in Saudi Arabia;<br>(b) The biological characteristics of the absentees;<br>(c) The spell and duration of sickness absence in different occupational groups and;<br>(d) the-major diseases requiring sickness absence	All employees at King Khalid University Hospital	Sickness absence records were reviewed for all employees of King Khalid University Hospital, Riyadh, Saudi Arabia over a 3 year period from 1989 to 1991.	There was a sickness absence rate of 7.7% with an average of 0.24 spells of sickness and duration of 1.24 days per absentee per year.<br>The biological characteristics of the absentees showed no differentials in the spell rate of sickness, but nurses and junior workers had higher duration of sickness absence than other categories of workers.<br>Acute respiratory and upper tract infections, sore throat, chickenpox, septicaemia, and diseases of the musculoskeletal system (mainly back pain) were the leading conditions resulting in absenteeism.
Becker and Oliveira 2008, Brazil	To research the rate of absenteeism of nursing professionals in a psychiatric center in Manaus, from January/2004 to January/2005, in the Human Resources sector of the institution	Nurses n=105	The records of workers who were absent from their professional activities for at least one day were checked in the study period	There were 415 absence reports in the nursing team during the studied period, by 74.29% of nursing professionals. The average rate of absenteeism is 2.79. The main reason reported for absenteeism was illness
Beil-Hildebrand 1996, Review of literature	To extend nursing managements' understanding of the topic (absenteeism) as a precondition for well-balanced schedules and effective human	Nurses	Use of previous articles to bring about an understanding of absenteeism as well as hypothetical examples.	Taking absence into account will have significant implications for a more balanced organization as well as for the quality and continuity of nursing care provided.

	resource planning.			Outlines various definitions of absenteeism as defined by other authors
Borda and Norman 1997, Malta	To test a model of absence and turnover of registered nurses developed from a review of the research literature (Gauci Borda and Norman, 1997)	Nurses (n=254)	Comparing the relationship between job satisfaction, kinship responsibility, pay, employment opportunity, intent to stay in employment and absence for male and female registered nurses.<br>Survey by questionnaire and absence data obtained from the hospital personnel records	A response rate of 67% (N = 171) Nurses satisfied with their job and intended to stay in their present employment for the next 12 months.<br>Job satisfaction associated with intent to stay for male nurses but not for females and kinship responsibility and intent to stay was associated with absence in female but not male nurses. An association was found between job satisfaction and absence. Several variables, including fear of change, which may influence intent to stay of Maltese nurses, were identified.
Boumans 1993, Netherlands	To examine the effect of two leadership styles on nurses reactions to their work situation Does need for autonomy among the nurses play a moderating role?	Nurses from 16 general hospitals n=561	Questionnaire survey asking about leadership style of head nurses and reactions of interest	96% response rate.<br>The more social leadership is displayed, the more job satisfaction<br>and meaningfulness and the fewer health complaints<br>As to instrumental leadership, a significant relationship only occurs with health complaints the more instrumental leadership is shown, the more health complaints.<br>Neither style is correlated with frequency of absence<br>Impossible to indicate one optimum combination of leadership styles to reduce absence frequency. Low absence frequency results from various leadership styles combinations
Burke 2000, Canada	To examine the effects of hospital restructuring and downsizing on nursing staff	1362 of 3900 nurses (full and part time)<br>About ½ full time	Questionnaire survey.<br>Measuring various effects of restructuring among them work	Full-time employees more likely to be absent and less likely to quit.

			outcomes (Job satisfaction, intent to quit and absenteeism)	
Chaudhury and Hammer 2004, Bangladesh	To quantify the extent of absenteeism among medical staff on a nationally representative scale absolute.	Doctors, paramedics, family welfare visitors, lab technician and pharmacist – No numbers given	A survey was conducted where unannounced visits were made to a sample of 180 health facilities with the aim of discovering what fraction of medical professionals were present at their assigned post.	Absentee rates do not show any significant difference between poor and rich areas. Vacant positions sometimes filled in by subs while in other cases no one performs duties of the vacant position.<br>Greatest proportions of vacant posts are those for doctors, pharmacists and senior Family Welfare Visitors relative to nurses, paramedics and junior FWV's.<br>For the whole sample of providers, the absentee rate is 35%.
Chaudhury, Hammer et al. 2006, Bangladesh, Ecuador, India, Indonesia, Peru and Uganda	A survey where unannounced visits were made by enumerators to primary schools and health clinics in Bangladesh, Ecuador, India, Indonesia, Peru and Uganda focusing on presence of teachers and health works.	Health care workers and teachers	Data based on direct physical verification of provider's presence.	19 percent of teachers and 35 percent of health workers were absent.<br>Absence rate higher in rural areas.<br>Absenteeism higher among doctors and headmasters.<br>Little evidence that pay affects absenteeism.<br>Working conditions affect absenteeism.
Carosi 2009, Canada	To identify factors that may predict a high frequency of sickness absenteeism in the workplace	Cancer care workers (clinical and non-clinical) n=244	Retrospective analysis of paid and unpaid leave, demographics, occupation and length of employment	Age, sex, job level, and duration of employment significantly associated with high sickness absenteeism<br>Full-time workers more likely to demonstrate a high frequency of sickness absence than were temporary workers; permanent workers were more likely to be absent than were temporary workers; and clinical and administrative employees more likely to be absent more frequently than were nonclinical employees (no statistical significance)

				Younger employees (less than 40 years of age) had a risk of high sickness absence that was significantly increased compared with the risk for older employees<br>Increased risk of high sickness absence observed for low job level workers as compared with high job level workers<br>Men less likely to be absent than women
Davey, Cummings et al. 2009, A systematic literature review	To identify and examine predictors of short-term absences of staff nurses working in hospital settings reported in the research literature	Nurses	Systematic lit review	Most common measure of absenteeism reported was absence frequency. Other measures are total days, percentage and duration.<br>Use of theoretical frameworks to conceptualize absenteeism
Donovan 2008, Canada	To merge Occupational Health (OH) and Human Resources (HR) administrative data to describe reasons for absenteeism among hospital employees	Hospital employees who have the ability to acquire or transmit infectious agents during the course of their work (physicians, nurses, therapists, cleaning/maintenance staff, patient care assistants, clerks, and technologists) n=1964	Retrospective cohort linking records in administrative databases.<br>Reasons for absence self-reported over telephone conversations	Most common self-reported reasons for absence were respiratory, gastrointestinal illnesses, and musculoskeletal injuries/ disabilities.<br>Employees working in the Department of General Medicine experienced the highest number of times absent.<br>The department with the highest percentage of staff not reporting to OH was General Medicine.
Eriksen 2003, Norway	To identify the work factors that predict sickness absence in nurses' aides	Nurses n=4931 of 5563	Prospective design.<br>Questionnaires filled twice 3 months apart.	Sickness absence more common in respondents who reported health complaints at baseline, smokers, those with low personal engagement in the work unit, lack of encouraging and supportive culture in the work unit, perceived unsuspicious culture in the work unit, working in psychiatric and paediatric wards, having injured the neck in an accident, having changed work or work tasks because of pain,

				musculoskeletal pain, and fatigue<br>Responders who engaged in aerobics or gym less likely to be absent from work.
Franche 2011, Canada	To evaluate the impact of worker and workplace factors and of their relationships on work absence duration	Female nurses engaged in direct care n=11762 of 15167	Structural equation modelling (a telephone survey completed)	Worker health factors, namely pain related work interference, work-related pain, and depression, had the largest total effects on work absence duration.<br>Emotional abuse or physical assault from a patient or visitor had the largest total effect on work absence duration of all workplace factors<br>Low respect and support at work and abuse or assault by a co-worker.
Fujishiro 2011, Philippines	To examined whether the experience of workplace aggression was associated with poor self-rated health, as well as with work-related injury or illness, and whether being exposed to physical assault and verbal aggression simultaneously was associated with a higher risk of work-related injury or illness, compared with being exposed to either type of aggression alone.	Nurses attending the 2007 national convention of the Philippine Nurses Association n=690 of 1000	Self administered questionnaire. Cross sectional design as the nurses were from 13 regions of the Philippines	Those who reported experiencing physical assault were more likely to have missed more than 2 workdays in the past year.<br>Targets of verbal abuse showed a trend toward missing work
Garcia-Prado and Chawla 2006, Costa Rica	To test whether the introduction of these new organizational arrangements was followed by lower absence rates than those registered previously in Costa Rica	Physicians and healthcare workers in 29 public hospitals.	Data on health care personnel absence, specifically cumulative sick-leave days were obtained. Average absence figures were then computed for each hospital and year by dividing total days of absence by total number of health staff.	Reforms had a negative impact on absenteeism, which increased throughout the considered period.<br>The policy of not substituting absentee workers, which was introduced through the reforms, did not work as expected in a permissive environment in which peer pressure mechanisms were lacking.

Gaudine and Gregory 2010, Canada	To determine the accuracy of nurses' self-reports of absence	Nurses n= 215	The study design is correlational and descriptive.<br>Self-reported absence from questionnaires completed nurses then compared with organizational records.	Strong positive correlation, a strong intra-class correlation and Cronbach's alpha for the two measures of absence. Difference in central tendency that is related to the majority of nurses in this study underestimating their days absent from work.
Gorman, Yu et al. 2010, British Columbia, Canada	To determine the demographic and work characteristics of healthcare workers who were more likely to take sickness absences from work.	Healthcare workers (n= 36,858). In 3 health authorities.<br>59.2% full-time and 40.8% part-time employees	Cross-sectional study design. Payroll data were analyzed for three health regions. Sickness absence rates were determined per person-year and then compared across demographic and work characteristics using multivariate Poisson regression models. The direct costs to the employer due to sickness absences were also estimated.	Female, older, full-time workers, long-term care workers and those with a lower hourly wage were more likely to take sickness absences and had similar trends with respect to the costs due to sickness absence. For occupations, licensed practical nurses, care aides and facility support workers had higher rates of sickness absence. Registered nurses, and those workers paid high hourly wages were associated with highest sickness related costs.
Genevay 2011, Switzerland	To explore the staff- and work-related risk factors for spinal pain among hospital employees,<br>To investigate the effect of staff- and work-related variables on the consequences of spinal pain, such as doctor visits and sick leave	Employees in a hospital n=1298 of 2700	Questionnaire survey.<br>Besides demographic factors also asked about spinal pain, work conditions and sick leave as a result of spinal pain	Work absenteeism - 15.7% , mean number of 21.7 days per year.<br>Physicians had the lowest rate of absenteeism and shorter duration of sick leave. Nurse assistants reported the highest proportion of work absenteeism and the longest duration of sick leave.<br>Frequency of sick leaves was related to age, sex, BMI, being a supervisor and number of days with pain
Gimeno 2007, Costa Rica	To assess the relationship of occupational and organizational risk factors with work-injury absence	Public hospital employees n=842/859	A cross-sectional survey.<br>Questionnaire with sociodemographic, occupational and organizational factors	General service employees at a higher risk of work injury absence compared to professionals.<br>Exposures to chemicals and radiation related to work injury absence.<br>Job task interference with safety

				practices and low level of safety practices associated with work-injury absence.
Isah, Omorogbe et al. 2008, Nigeria	To determine the overall absenteeism rate and the factors that conduces to absenteeism among hospital workers.	Entire staff in the hospital (n=474)	Cross sectional descriptive type. Data were collected through self-administered and semi-structured questionnaire.	53% - at least one absence spell in the year. Absenteeism was highest among domestic staff and lowest among the doctors. Average number of spells per absentee per year - 1.7, average duration of spells was 4.1 days, incapacity rate - 7.2 days. Overall absenteeism rate - 1.7%.
Johnson, Croghan et al. 2003, Lit Review - UK	To examine the accumulation of research based knowledge to provide a healthcare perspective on the problem of sickness absence management.	Health workers	A Literature review was carried out to providing a broad conceptual context by which the problem of sickness absence in the NHS can be examined.	Sickness absence management within the NHS is challenging but provides opportunities to improve the working lives of NHS employees. Sickness absence cannot be eradicated but it can be reduced by a selection of measures that reflect the uniqueness of the NHS. The many and diverse causes of sickness absence need acknowledgement, when devising strategies that can effectively provide solutions to the problems of sickness absence.
Josephson, Lindberg et al. 2008, Sweden	To ascertain whether the same individual factors, working conditions and health problems had led to increased probability of both leaving jobs and prolonged sickness absence in a cohort of Swedish nurses over a period of 3 years.	Nurses and assistant nurses (n=2293)	A baseline questionnaire was answered by 2293 nurses, representing a response rate of 86%. Exposed and unexposed nurses were compared with regard to two outcomes. During the 3-year follow-up, exposed and unexposed nurses were compared with regard to two outcomes: resigning and having at least one sick leave spell that lasted 28 days or longer.	18% of the nurses left their employment, and 16% had sick leave spells ≥28 days. Work in geriatric care, being socially excluded by superiors and/or workmates, negative effects of organizational changes and poor self-rated general health were factors that increased the likelihood of both leaving jobs and long-term sick leave.
Junkes 2010, Brazil	To demonstrate the scenario of	Nurses and physicians	Retrospective analysis of	Most common reason for health worker

	implicit payroll expenses caused by non-programmed absences justified by a medical declaration, lasting up to 15 days, without taking into account the description of the nursing professionals and physicians' disease	H1 n= (28 physicians, 69 nurses)<br>H2 n = (38 physicians, 37 nurses)	database.	absence was sickness.<br>The consequences of their absence considered is financial implications
Kalisch, Tschannen et al. 2011, USA	To examine whether actual nurse staffing predicts missed nursing care, controlling for other unit characteristics.	Nursing staff members with direct care responsibilities (n = 4288) on 110 care units.	The study utilized a cross-sectional, descriptive design.<br>The MISSCARE Survey was utilized to capture respondents' perceptions of missed nursing care as well as other unit characteristics	Hours Per Patient per Day was a significant predictor of missed nursing care ($\beta = -0.45$, $P = 0.002$).
Kivimaki, Elovainio et al. 2000, Finland	To investigate whether bullying in hospitals is associated with records of staff sickness absence.	Hospital staff (n=674 male and 4981 female employees)	The Poisson regression analyses of medically certified spells (≥4 days) and self certified spells (1–3 days) of sickness absence, relating to bullying and other predictors of health<br>Data on sickness absence gathered from employers' registers.	302 (5%) of the employees reported being victims of bullying.<br>Victims of bullying had higher body mass and prevalence of chronic disease, and their rates of medically and self certified spells of sickness absence were 1.5 and 1.2 times higher than those of the rest of the staff.
Kivimaki 2004, Finland	To examine whether primary nursing is associated with lower sickness absence rates than team nursing is	Nurses n= 1213<br>629 Primary nursing<br>613 Team nursing	Longitudinal cohort design.<br>Sickness absence records linked with information on the organization of nursing in the wards. 3 years follow up.<br>Sickness absence data obtained from records	No significant changes in absence rates during the follow up period<br>Primary nursing associated with 26–42% higher annual rates of short spells of sickness absence<br>Short sick leaves was 1.4 times higher for the primary nurses than for the team nurses in the second year and 1.6 times higher in the third year<br>Turn over higher in primary than in team nursing
Kivimaki, Sutinen et	To identify determinants of	Hospital Physicians	The Poisson regression analyses	Physicians took one third to a half the

al. 2001, Finland	sickness absence in hospital physicians.<br>To examined the extent to which factors related to health, work, and social circumstances are associated with recorded sickness absence among physicians.	(N=447)<br>Controls female head nurses and ward sisters (n=466)	of short (1–3 days) and long (>3 days) recorded spells of sickness absence relating to potential determinants of sickness absence were based on a 2 year follow up period	sick leave of controls.<br>All the health outcomes were strongly associated with sickness absence in both groups.<br>Teamwork had the greatest effect on sickness absence in physicians.<br>Physicians working in poorly functioning teams were at greater risk of taking long spells.<br>Risks related to overload, heavy on call responsibility, poor job control, social circumstances outside the workplace, and health behaviors were smaller.
Kristensen 2010, Denmark	To examine differences in sickness absence among occupational groups in a large general hospital	Workers in a hospital n=2331	Prospective cohort – analyzing records data.<br>Social economic status accessed by job title	Women had more of all types of absences than men.<br>"Normal" absence increased with age and "abnormal" absence decreased with age<br>Doctors and physiotherapists had fewer absence compared to other groups<br>Sickness absence increased with decreasing socioeconomic status.
Libet 2001, USA	To examine the relationship between employee absenteeism and productivity in an outpatient mental health clinic	Clinicians at a Veteran Affairs (VA) Medical Center outpatient mental health clinic n=22	Examination of archival absence data for the previous year.	There was a significant inverse relationship between sick leave maximum and absenteeism on Mondays and Fridays and long-term absenteeism patterns<br>Workers that take sick and family leave are not necessarily unproductive.
Lim 2002, Thailand	To compare sickness absence among hospital personnel with different work types	All personnel in a university teaching hospital n=2181	Descriptive analytical study of longitudinal data	Laboratory personnel had much higher short-term absence than doctors.<br>Nurses more absent than doctors.<br>Nurses and doctors had lower absence than other categories.<br>Less variation in long-term absence.<br>Doctors had lowest while manual

				workers had highest.<br>Short term absence higher in males, married employees and older employees
Matrunola 1996, UK	To consider the factors which affect job satisfaction, and the relationship between these and the individual's rate of absence	Nurses n=50	Interviews first conducted with 6 randomly selected nurses in different grades in order to inform what to be included in questionnaires.<br>The validated questionnaires later used to collect data on burnout and satisfaction.<br>Absence data collected from records of the previous year.	Little support for the hypothesis that there is a relationship between job satisfaction and absenteeism<br>An inverse relationship between age and absence are noted<br>Absenteeism may not be a sensitive indicator of job satisfaction<br>Job satisfaction is a reliable indicator of burnout
Manzi 2012, Tanzania	To report on staffing levels and productivity in peripheral health facilities in southern Tanzania	Nurses and Clinical staff	Health facility survey conducted. Those absent identified and reasons for absence sought	44% of clinical staff absent. 49% of nurses absent.
Muthama, Maina et al. 2008, Kenya	To evaluate the rates of absenteeism of health providers in the health sector	Health workers n=497	Physical verification of absence of health workers. Reasons for absence recorded	Reasons reported for absence: Family /personal issues like funeral/ to collect salary, Late and late from lunch, maternity/ annual leave, night off, official (training, workshop), off duty, sick off, no reason given (49.7%)<br>Demographics. Consequences also reported
Ng_2009, Hong Kong	To estimate the prevalence of workplace violence involving radiographers in Hong Kong, to evaluate underlying factors contributing to incidents and their impact and to suggest improvements in management and training	Radiographers n=150 of 281	Questionnaire survey. The radiographers were from different hospitals	61% had experienced violence<br>Work stress, job dissatisfaction, depression, lack of willingness to work, increased sick leave.
Nyathi 2008, South Africa	To explore and describe the working conditions that contributes to absenteeism	Professional and Sub-professional nurses n=233 of 270	Questionnaire survey collecting data on demographics, nurses' characteristics, characteristics of	Working conditions that contribute to absenteeism.<br>Inadequate group cohesion, inadequate

	among professional and sub-professional nurses at a provincial hospital		the manager, work characteristics and organization characteristics.<br>The current article is on work characteristics	delegation of autonomy, role ambiguity, ineffective routinisation and the effect of workload in the workplace
Peterson, Bergstrom et al. 2011, Sweden	To investigate the predictive validity of the Oldenburg Burnout Inventory (OLBI), the Hospital Anxiety Depression Scale (HAD), and of self-rated health (SRH) on future long term sickness absenteeism. (LTSA) (90 days or more).	Female health professionals	Survey. Self administered questionnaires.<br>Long-term sickness absence data (90 days or more) were collected from the insurance company that supplies complementary sickness insurance for all County Council employees in Sweden.	High scores on exhaustion, depression, and poor self-rated health increased the risk of future LTSA (≥90 days).
Plant 2003, UK	To discover which factors were considered relevant by practice and community nurses to the decision to take sick leave	Nurses n=9 of 50	Semi-structured **in-depth interviews** at respondents' time and location of choice.	Practical considerations of taking sick leave (cause and consequence of taking sick leave), psychological issues felt by those that remain (stress, burnout and feelings of guilt.), workload, guilty of taking sick leave.<br>Personal and professional values – more on causes of presenteeism (guilty of taking sick leave), avoiding infecting patients. Management of sickness absence (lack of substitutes), work characteristics (Control of demand, control of workload and expectation of support from management and colleagues).<br>Individual consideration (individual health status and pattern of sick-leave taking, as well as the influence of family commitments).
Pinar 2011, Turkey	To determine the perceived verbal and physical violence and related factors experienced by	Nurses n=255 of 262	Questionnaire data	Frequency of verbal violence 91.4%, 79.4% of nurses exposed to physical violence, perpetrators of violence are

	nurses in emergency settings.			patients' relatives and patients.<br>Most nurses reported feeling fear, 3% took sick leave and 80% did not report the incidence.<br>Most sought support from colleagues rather than the administration
Pompeii 2010, USA	To examine personal and workplace characteristics as possible predictors of losing time away from work among nurses and nurses' aides who sought treatment for work related back pain	Nurses and nurses' aides n=589 of 709	Prospective cohort (All work-related back pain claims were identified through first report of injury data from 1994 through to 2006<br>Those identified were included in the survey and filled the questionnaire on the day of first visit<br>Data on absenteeism obtained from physicians recommendations)	Back pain most likely to result from patient handling activities, handling of equipment, and slips, trips, and falls.<br>High physical demands reported.<br>Lost workdays incurred by 181 (30.7%) nursing personnel, with n = 112 incurring ≤7 while n = 69 lost ≥8 days<br>Most of the workplace characteristics failed to predict the overall occurrence of lost workdays regardless of the duration of time lost.
Pines, Skulkeo et al. 1985, Israel	To identify according to some demographic (sex, age, marital status) and occupational (occupational group, duration of hospital employment) variables those groups of hospital employees who are at higher risk of sickness absenteeism.	Doctors n= 623 (medical doctors and dentists, nurses n=843 (qualified and practical nurses and nurses' aides), technicians n=414 (radiological, ECG, EEG, laboratory, and other), administrative and clerical n=405 workers, skilled workers 146, unskilled workers 431 and other occupations 144.<br><br>n= 3020	Study was based on the sickness absence records routinely completed in the personnel and occupational health departments of the Jerusalem Hadassah Hospital for 1981.<br>The study population was 3020 employees (the total staff of the Ein Karem Hadassah Hospital, Jerusalem) was distributed according to the demographic and occupational variables.	During the study period (1981)8146 reported spells of absence with 34 829 days lost were attributed to sickness.<br>A comparison with the data of the study performed in the same hospital about 15 years ago showed a rise in the duration of absences with a simultaneous reduction in their incidence.<br>Sickness absenteeism was higher among female, mainly unskilled, workers, presently or previously married, aged from 45 to 60, and employed in the hospital for over ten years.
Rajbhandary 2010, Canada	To investigates the relationship between the working conditions and illness- and injury-related	Registered and licensed practical and registered psychiatric nurses n=	Data extracted from a national survey of the work and health of nurses i.e including absence data	The average absenteeism for RNs is less than those for LPNs.<br>Role overload score and effort–reward

	absenteeism among nurses	18,676 of 23428<br>After restricting the sample to fulltime employed RNs and LPNs and eliminating those with missing data total participants n=6634		imbalance score significant for LPNs but not for RNs.<br>The province in which one works influences their absence rates.<br>Those working in Newfoundland and Those working in hospitals have higher absence rates.<br>Females more absent than males.
Rauhala, Kivimäki et al. 2007, Finland	To examine whether nurses' work overload is associated with increased sick leave and quantifying the loss of working days from work overload.	Nurses (registered and practical) n=877	An observational cohort study Patient-associated workload scores from the RAFAELA system were based on a 6-month monitoring period in 2004. Records of 12-month sick leave in the same year were obtained from employers' registers.	The mean workload was 9% above the optimum. There was a linear trend between increasing workload and increasing sick leave. Among nurses with workload ≥30% above the optimum the rate of self certified periods of sick leave was 1.44 times higher than among those with an optimum workload.<br>The corresponding rate ratio for medically certified sick leave was 1.49 These excess rates of sickness absence resulted in 12 extra sick leave days per person-year.
Remsburg, Armacost et al. 1999, USA	To identify reasons for absenteeism among nursing assistants.<br>To identify the impact that absenteeism had on achieving daily target hours of care.<br>To identify strategies to reduce absenteeism.	Nurse Assistants in Long-term care units	The following data were collected: (1) number of daily call-ins, (2) reason for call-in, and (3) the percentage of shifts not meeting daily targeted hours of care.	A total of 300 call-ins during the 3-month period. Only 6 days during the 3-month period had no call-ins. Number of daily call-ins ranged from 0 to 9, with a mean of 3.3 call-ins per day. Approximately 79 nursing assistants were assigned for duty each weekday; therefore, the average rate of absenteeism was 4%.
Ritchie, Macdonald et al. 1999, UK	To identify the healthcare needs of healthcare workers using routinely collected sickness absence data	Nurses, ancillary, administrative and clerical staff, and medical and dental staff (n=12900)	Analysis of sickness absence records for one year - 1993	Female staff more likely to have experienced absence than male staff.<br>Full time staff had greater rates of sickness absence than part time staff.<br>Medical and dental staff had the lowest rate of recorded sickness absence.

				Maintenance staff had proportionately the greatest number of absences of >1 week and pharmacy staff had the fewest of these longer term absences.<br>The main known causes of absence were respiratory disorders, digestive disorders, and musculoskeletal disorders.
Schalk 2011, Netherlands	To examine the influence over time of organizational commitment, health complaints, and visits to a general practitioner on sickness absenteeism.	Nurses n= 224	A longitudinal, three-wave study in two nursing homes.<br>Questionnaire data (self reports of organizational commitment, health complaints, visits to a general practitioner)<br>Absenteeism data retrieved from personnel files.	Health complaints and visits to a general practitioner were found to predict absenteeism behavior. Commitment was related to health complaints at the same point in time, but did not predict future sickness absenteeism.
Schreuder, Roelen et al. 2011, Netherlands	To investigate nurse managers' leadership behavior in relation to the sickness absence records of nursing staff.	Nurses n = 699 (664 female and 35 male nurses)	Cross-sectional study design<br>Individual sickness absence records were linked to the nurse managers' leadership.<br>SPSS used for analysis	The nurses had an avg of 65.8 days of sickness absence during the study period and an avg of 4.0 episodes.<br>Leadership flexibility of nurse managers was not associated with sickness absence among nurses. High leadership effectiveness was associated with fewer days and fewer short episodes of sickness absence. Leadership effectiveness was unrelated to the number of long episodes of sickness absence.
Schreuder, Roelen et al. 2010, Netherlands	To investigate health and work factors in relation to the frequency of short-term sickness absence among nurses	Nurses n=328 of 459	Cross sectional study using self administered questionnaire to measure general and mental health, job demand, support and control, work efforts and rewards and over commitment.<br>Questionnaire data then linked to absence data for preceding	Age, general health and over-commitment inversely related to number of short sickness absence episodes.<br>Workplace social support and ER ratio inversely associated to long and short sickness absence episodes respectively<br>MHI positively associated with number of short sickness absence episodes

			3years	
Schreuder, Plat et al. 2011, Netherlands	To investigate the relationship between coping styles and sickness absence in healthcare	Nurses n=386 of 566	Prospective cohort. A baseline questionnaire on coping styles was used. This was then matched to the sickness absence prospectively for one year.<br>Health status, work efforts and work rewards were included in the baseline questionnaire<br>Age and number of hours worked were obtained from records	Problem-solving coping negatively associated with the number of long episodes of sickness absence<br>Social coping negatively associated with the number of both short episodes and long episodes of sickness<br>Palliative avoidant coping not associated with sickness absence among female hospital nurses<br>After adding the health status to the Poisson regression models, the associations between social coping and sickness absence lost statistical significance
Siu 2002, Hong Kong	To investigate the role of organizational climate and psychological distress on job satisfaction; and the role of climate, distress and job satisfaction on absenteeism	General and psychiatric nurses<br>Sample 1 n=144 of 144<br>Sample 2 n=114 of 200	Cross sectional design. Nurses filled in self administered questionnaire 8 months apart measuring organizational climate, psychological distress, job satisfaction and absenteeism	Age and type (general or psychiatric) were statistically significantly correlated with absenteeism for the first sample, and type with absenteeism for the second sample<br>Absenteeism was negatively related to job satisfaction and positively related to psychological distress for the first sample only<br>Well-being appeared to be a significant predictor of job satisfaction
Song, Daly et al. 1997, USA	To examine the effectiveness of a nurse-managed special care unit (SCU) for chronically critically ill patients, developed to improve patient outcomes as well as nurse outcomes.	Nurses n=143 of 208	Comparative study between two nursing units under two different models of practices.<br>An annual survey was conducted among nurses in the two units. Study lasted 4 years.	The SCU nurses reported greater job satisfaction with payment and supervisory dimensions than the ICU nurses.<br>The percentage of absent hours for nurses in the SCU was significantly lower than that for nurses in the ICUs
Steenstra 2005, Netherlands	To describe the course of sick leave due to LBP in a cohort of workers absent from work for 1	Health care professionals who reported sick leave due to nonspecific LBP	26 week prospective cohort study. After calling in sick, workers were obliged to fill in a	Median time RTW was 5 days, median time LRTW was 6 days, median number of TDSL during follow-up was 6 days.

	day or more due to non-specific LBP.<br>To determine prognostic factors for duration of sick leave due to LBP.	for more than 1 day over a 2-year period<br>n=615	sick leave questionnaire on the nature and expected prognosis of the sick leave episode.	Self-reported expected duration of sick-leave, treatment by GP, seeking OP care, and the self-reported possible causes of sick-leave, diminished mobility, being admitted to hospital and function classification: maintenance, were univariately associated with both RTW and LRTW<br>Complaints due to other reasons was univariately associated only with RTW
Trinkoff, Storr et al. 2001, USA	To examined associations among eight physical demands and inadequate sleep, pain medication use, and absenteeism	Registered Nurses<br>n=3727 of 5706	Cross –sectional survey. Data were collected with an anonymous mailed survey. Those in the sample received up to six contacts, including three copies of the questionnaire.	17% used at least one sick day in the last month. Potential consequences of physical demand; inadequate sleep, pain medication use, absenteeism.<br>As the number of demands increased, the likelihood of each outcome increased.
Tripathi, Mohan et al. 2010, India	To assess the problem of leave of absence among nurses at a tertiary care hospital in India	Nurses n=385 of 550	Retrospective analysis of absence data from leave records (1 year)	68.9% of nurses took leave at least once during the year.<br>Average sickness leave 11.2 days/person<br>Non-sickness leave of 16.2 days/person.<br>Sickness leave rate 8.82 days/ spell<br>Non-sickness leave rate 10.4 days/spell<br>Ward nurses took the highest number of unplanned sickness leave rate; planned sickness leaves were highest among those in operation theatres and ICU.<br>Planned sickness leave was highest among younger nurses in operation theatres and intensive care units.
UNHCO 2010, Uganda	To document the prevalence, determinants and consequences of absenteeism of health providers in the health sector of Uganda	Health facilities n=78<br>Health workers in the 78 facilities n=177	Review of literature, individual interviews, attendance tracking – 7 unannounced visits, key informant interviews and focus group discussions	Average rate of absenteeism 47.9%.<br>Males more absent<br>Determinants of absenteeism: work environment, supervision, Job satisfaction with different incentives, level of education, and location of health

				facility (remoteness), length of service in the current facility, job stress, and Marital Status
Unruh, Joseph et al. 2007, USA	To assess the impact of nurse absenteeism on the quality of patient care	Nurses	A retrospective study was carried out using unit-level data from incident reports and staffing records for 2004. Records were checked monthly for a year in 6 units hence there were 72 observations	Nurse absenteeism was related to lower use of alternatives to restrain.<br>A combination of high RN absenteeism and high patient load was related to greater use of restraints and a higher number of deaths.
Van Wyk 2010, review of literature	To assess the effects of preventive staff-support interventions on health workers	Healthcare workers	Systematic review of literature Randomised controlled trials of interventions to support healthcare workers in coping with work-related stress, preventing burnout<br>and improving job satisfaction	No study assessed the effects of support groups for health workers.<br>Eight studies assessed the effects of training interventions in various stress management techniques on measures of stress and/or job satisfaction, and two studies assessed the effects of management interventions on stress, job satisfaction and absenteeism.<br>Management interventions demonstrated increases in job satisfaction, but failed to show effect on absenteeism.
Verhaeghe, Mak et al 2003, Belgium	To examine differences in stress perception by middle-aged HCW and controls working in the Flemish part of Belgium.<br>The relationship between job stress perception & absenteeism was studied prospectively.	HCWs n=315 (55 men & 260 women)	Case control study with data based on data from the Belgian cohort study of the relation between job stress and health called 'BELSTRESS'<br>Sickness absence is measured in the year following the interview	HCW score higher for perceived psychological workload but they experience less decision latitude in comparison with controls.<br>For social support no significant differences were found.<br>Among HCW there was a positive correlation between incidence of sickness absence and 'job demand'. The opposite was found among the controls<br>For 'decision latitude' HCW reported in the highest quartile reduction in the odds of sickness absence. Controls showed a negative correlation between

				'decision latitude' and sickness absence.<br>Strong negative correlation between the incidence of sickness absence and 'social support' for HCW.<br>Belonging to the 'job–strain' population increased risk of being absent
Verhaeghe, Vlerick et al. 2006, Belgium	To study how the occurrence and appraisal of recurrent changes (supervisor, tasks, colleagues, working hours, location) in the work environment of hospital nurses affect psychological well-being (i.e. job satisfaction, eustress and distress) and absence through illness.	Nurses n=2094	Cross-sectional research design. Absenteeism was measured in the 12 months following the questionnaire (measuring stress and satisfaction)	Lower rates of sickness absence and higher levels of job satisfaction and job stress among male nurses.<br>Nurses confronted with changes in the past 6 months noted higher levels of distress, no relation to job satisfaction and eustress, or to absenteeism.<br>Strong association btn 'challenge' and job satisfaction, and eustress - no relationship with distress – no association sickness absence.<br>The lower the level of 'threat', the higher the level of job satisfaction and eustress.<br>Positive association between the incidence of sickness absence and the level of 'threat' did not differentiate btn public and private
Wier 1997, Canada	To report on a randomized controlled trial that was designed to assess the potential for leadership effectiveness in improving staff morale and quality of care as well as its effect on the cost of nurse absenteeism.	Nurses and nurse managers	Consultative meetings with nurse leaders with the intention of influencing their leadership style and in return influence the absence rates of their teams.	Improvement in the characteristics of their work setting and in the quality of working relationships, no statistically significant difference in absenteeism,
Williams, Rondeau et al. 2007, Canada	To see the impact that heavy workloads directly have on physician stress and more distally on physician job	Physicians n=480	Initial survey was mailed to all physicians followed six weeks later with a second survey mailing.	Workload related to perceived level of Stress. Stress was negatively related to job satisfaction<br>Job stress was found to be significantly

	satisfaction, job performance, patient care quality, absenteeism turnover intentions and organizational performance.		Structural equation analysis with latent variables (LISREL 8) was used to test the model.	related with absenteeism, while job satisfaction was not.<br>The relationship between workload and absenteeism suggests the notion that as workload increases, absenteeism increases.
Wilson, Diedrich et al. 2011, USA	To determine the degree of HH in the workplace and the extent that the perception of HH affect ill calls and the likelihood of leaving their current position	Nurses n=130	Retrospective descriptive cross-sectional design	83.1% had seen HH. 95% believed that HH contributed to ill calls but only 20% admitted to having called in sick because of HH.<br>19% were definitely going to leave their current position and 20.5% were considering leaving their current position because of HH.
